# Extracorporeal membrane oxygenation (ECMO) in patients with tuberculosis: systematic review and meta-analysis of 43 cases

**DOI:** 10.1186/s12890-023-02715-x

**Published:** 2024-01-22

**Authors:** Raja Idris, Ann-Sophie Zielbauer, Julia Koepsell, Jan Kloka, Nils Wetzstein

**Affiliations:** 1 Department of Internal Medicine, Infectious Diseases, Goethe University Frankfurt, University Hospital, Theodor-Stern-Kai 7, 60590 Frankfurt, Germany; 2 Department of Anaesthesiology, Intensive Care Medicine and Pain Therapy, Goethe University Frankfurt, University Hospital, Frankfurt, Germany; 3 Molecular and Experimental Mycobacteriology, Research Centre Borstel, Borstel, Germany

**Keywords:** TB, Tuberculosis, Extracorporeal membrane oxygenation, ECMO

## Abstract

**Introduction:**

Tuberculosis (TB) is still a major contributor to the global health burden. Pulmonary TB can lead to life-threatening respiratory failure necessitating extracorporeal membrane oxygenation (ECMO) therapy. However, data on ECMO experience in the management of TB patients are scarce.

**Methods:**

We conducted a systematic review of the literature using the search terms *ECMO, extracorporeal membrane oxygenation, TB* and *tuberculosis* in three databases (Medline, Web of Science and EMBASE). Clinical data were extracted by two independent investigators. Clinical parameters, such as mode of ECMO therapy, duration of treatment and clinical outcomes, were assessed.

**Results:**

Overall, 43 patients from 15 countries were included in the analysis. The age ranged from 0 to 65 years, 39.5% were male, and 60.5% were female. The majority of patients suffered from ARDS (83.4%), with a mean Horovitz quotient of 68.1 (range 30.0–131.0). 83.7% received VV-ECMO, and 24.3% received VA-ECMO. Coinfections and complications were frequently observed (45.5% and 48.6% respectively). At the end of the respective observation period, the overall outcome was excellent, with 81.4% survival.

**Discussion:**

ECMO therapy in TB patients appears to be a feasible therapeutic option, providing a bridge until antimycobacterial therapy takes effect. As the underlying cause is reversible, we advocate for the evaluation of ECMO usage in these patients with acute cardiac or respiratory failure.

**Supplementary Information:**

The online version contains supplementary material available at 10.1186/s12890-023-02715-x.

## Introduction

Tuberculosis (TB) is still a major contributor to the global health burden. After COVID-19, TB is the deadliest infectious disease from a single agent [1]. In 2021, there was an upsurge in the estimated number of deaths from 1.4 million in 2019 to 1.6 million [1]. The gap in access to TB diagnosis and treatment during the COVID-19 pandemic will continue to negatively impact the WHO’s END TB strategy for years to come [2].

Nevertheless, the COVID pandemic also brought a variety of new insights, for example into the treatment of acute respiratory distress syndrome (ARDS) [3]. According to the Berlin definition “ARDS is a type of acute diffuse, inflammatory lung injury, leading to increased pulmonary vascular permeability, increased lung weight, and loss of aerated lung tissue” [4]. With the New Global Definition of Acute Respiratory Distress Syndrome, the Berlin Definition has been expanded, “including the use of high nasal oxygen (HFNO), expanding the use of pulse oximetry in place of arterial blood gases, use of ultrasound for chest imaging, and the need for applicability in resource-limited settings” [5]. Pulmonary TB, if left untreated, can also lead to life-threatening respiratory failure [6]. Nevertheless, ARDS secondary to tuberculosis remains a rarity: studies from South Africa and India estimated that 2 to 4.8% of ARDS cases admitted to the ICU were caused by tuberculosis [7]. The numbers for tuberculosis-related ARDS are probably lower in countries with a lower prevalence of TB.

Therefore, practical experience in the management of ARDS due to tuberculosis is still limited. For non-TB patients with severe ARDS, treatment by extracorporeal membrane oxygenation (ECMO) is a well-established treatment option. ECMO is a form of extracorporeal life support that utilizes a heart–lung machine to facilitate gas exchange and minimize ventilator-associated lung injury [8]. The number of ECMO runs and survival rates have been increasing within the last decade, with a worldwide overall survival rate of 67% in 2022, and for adult patients receiving ECMO for respiratory failure the survival rate was 66% [9]. Moreover, it has recently been shown that 90-day mortality for patients, fulfilling the American–European Consensus Conference definition or the Berlin definition for ARDS, is significantly lowered by venovenous ECMO compared with conventional ventilator management [10]. However, ECMO therapy is still associated with a variety of complications such as bleeding, embolisms or circuit failure [11]. However, mortality for TB patients who required intubation or ARDS treatment other than ECMO ranged from 62 to 69% [12, 13]. The use of ECMO treatment can lead to significantly improved survival rates in non-TB patients, but is rarely conducted in patients with TB [8].

Herein, we present the available data on ECMO treatment in TB patients to evaluate whether ECMO therapy is a viable option for these patients.

## Methods

This systematic review was performed in accordance with the current Preferred Reporting Items for Systematic Reviews and Meta-Analyses statement and checklist (PRISMA) [14]. A prespecified protocol for this systematic review was registered with the International Prospective Register of Systematic Reviews (PROSPERO CRD42022357405) on September 23rd 2022. We conducted a systematic review to identify studies about ECMO usage in the management of tuberculosis patients to investigate the efficacy of ECMO therapy within this cohort.

### Search strategy and selection criteria

The databases Medline (PubMed), Web of Science, and EMBASE were searched without any restrictions on year of publication and language on September 1^st^, 2022, February 14^th^, 2023, and September 20^th^, 2023 for eligible studies. The search strategy was developed by using a combination of Medical Subject Heading terms (MeSH) with the keywords “extracorporeal membrane oxygenation “, “ECMO”, “tuberculosis” or “TB”. As the same operators were applicable for all three databases, the final search term ("tuberculosis" OR "Tb" OR "TBC" OR "Tuberc*") AND ("extracorporeal membrane oxygenation" OR "ECMO") was applied. Duplicates were removed, and the remaining references were independently screened by two reviewers (RI and ASZ). For each study, titles and abstracts were screened for eligibility. Afterwards, all relevant full-text manuscripts were reviewed by the same two authors in regard to the inclusion and exclusion criteria. In case of multiple articles reporting on the same patient, the information from all of them was merged into one data set. Differences were resolved through discussion, and final decisions were made by vote after consulting with NW.

### Inclusion and exclusion criteria

Included were all studies reporting on a minimum of one patient with active/current tuberculosis caused by *Mycobacterium tuberculosis* complex and the use of ECMO therapy regardless of the patients’ age. The exclusion criteria were a history of previously treated and not currently active tuberculosis or mycobacterial infections due to non-tuberculous mycobacteria (NTM).

### Statistical analysis

Clinical data were independently extracted by two reviewers (RI and ASZ) and checked by a third investigator for plausibility (NW). We extracted demographic parameters, such as age, sex, country of treatment and origin, clinical manifestations of TB and comorbidities as well as microbiological parameters including drug sensitivity, antimycobacterial therapy, and coinfections. For the evaluation of ECMO treatment variables such as the Horovitz quotient, type of ECMO (venovenous (VV V-V) / venoarterial (VA V-A) / (venovenoarterial (VVA V-VA)), duration of ECMO therapy, the possible advent of complications, and whether the patients expired on ECMO were collected. For subsequent mortality analysis, the status of survival was evaluated at the respective time of publication. All data were collected into an Excel database, and missing data were handled as “NA” (not available) in the resulting data table.

All statistical analyses were performed in R V.4.2. (“Vigorous Calisthenics”) using the *tidyverse* [15]*.* Categorical data are depicted as nominators with denominators and percentages, and continuous data are depicted as the mean with range for normally distributed data and median with interquartile range for non-normally distributed data. Normality was tested using the Shapiro–Wilk-Test. Geographical data were depicted using the *ggmap* package within R [16]. The overall survival of the included patients was assessed by generating an unadjusted Kaplan–Meier-curve with time-to-event analysis in R’s *survival* and *survminer* packages [17, 18]. For all statistical tests a significance level of alpha = 0.05 was used.

## Results

### Included patients and general characteristics

**Fig. 1:**
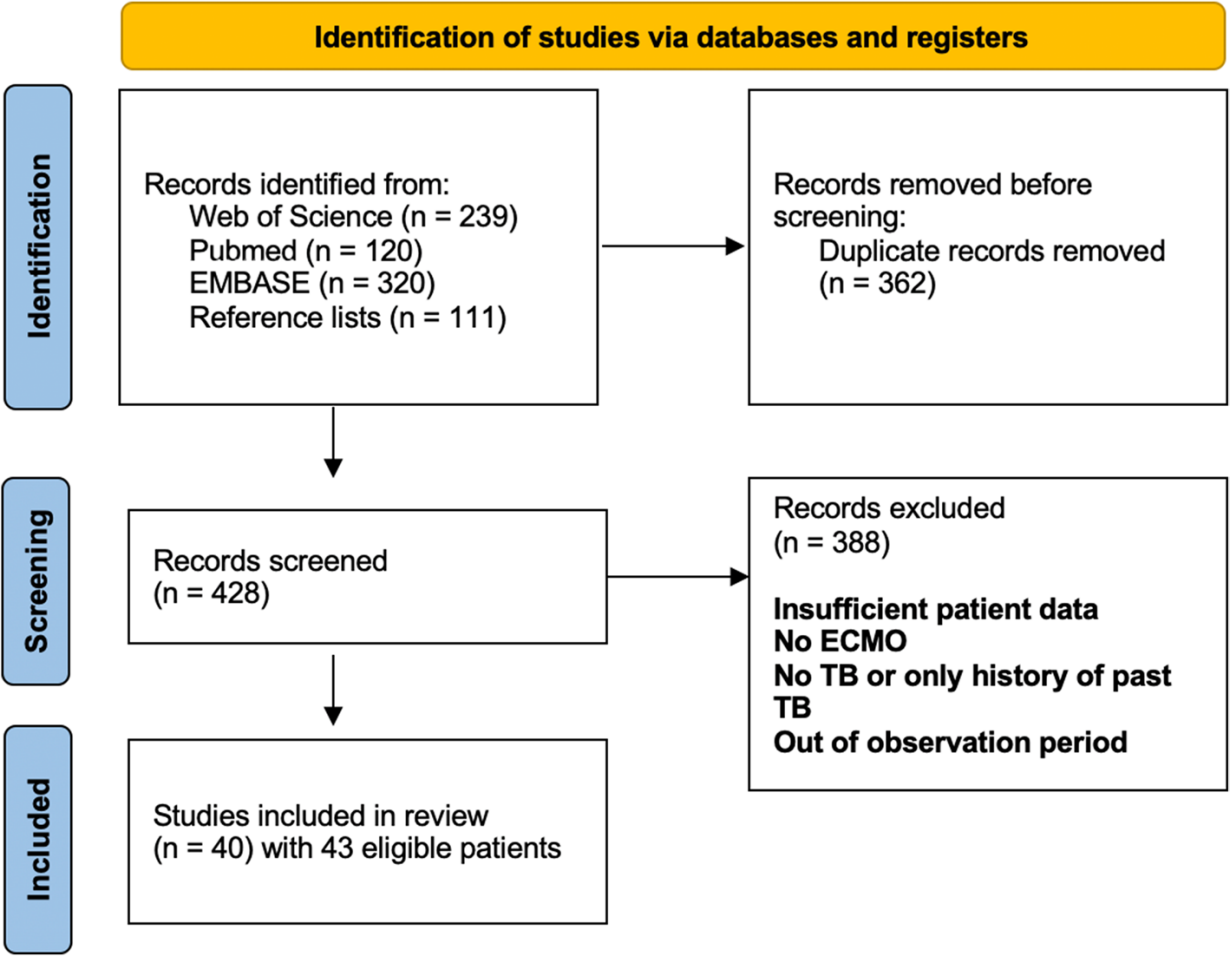
Flowchart of included articles and patients

**Fig. 2:**
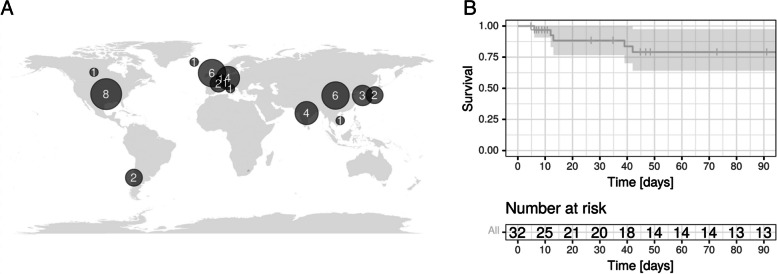
Geographical origin of patients (**A**) and overall survival probability for the first 90 days after implantation of ECMO (**B**)

Overall, 40 publications from 1975 to 2022 were included resulting in a patient cohort of 43 patients (37 case reports, whereby four case reports reported on the same two patients, one case series, one retrospective cohort study and one prospective cohort study) (Fig. 1). The country with the highest number of published articles was the United States with 8 cases, followed by China with 5 cases and Germany with 4 cases (Fig. 2A). The mean age of the patients was 29.5 years (range 0–65 years), 39.5% (15/38) were male, and 60.5% (23/38) were female (Table 1). The majority of patients did not have any comorbidities. However, six patients received immunosuppressive therapy, four suffered from pre-existing diabetes, one from an HIV- infection, and in five cases, the patients were pregnant. In 41 articles, the clinical manifestation of tuberculosis was described: 68.3% (28/41) of the patients had isolated pulmonary tuberculosis, 29.3% (12/41) suffered from a disseminated infection, and one patient had isolated extrapulmonary tuberculosis (pericardial manifestation). Thirty-six of the 43 patients (83.7%) had pulmonary involvement and eight (21.1%) had additional abdominal involvement. Nine patients (23.7%) suffered from additional other manifestations such as bone marrow or muscle abscesses. Most patients (63.3%, 19/30) presented with fever, followed by weight loss (36.7%, 11/30) and night sweats (13.3%, 4/30). In X-ray or computer tomography (CT) examinations, 29 out of 33 patients (87.9%) showed bilobal infiltration, and 39.4% (13/33) showed cavities. During the observation period, 15 of 33 patients (45.5%) developed a coinfection, 10 of whom (66.7%) had bacterial pneumonia, 2 (13.3%) had a bloodstream infection, and 3 (20%) had a fungal infection (one candidiasis, one mucor-infection and one aspergillosis) (Table 1).

**Table 1:** Baseline characteristics of included patients

	**n**	**data available**	**[%]**
**Age (mean, range)**	29,5 (0 – 65)	34	
**Gender**			
Male	15	38	39.5
Female	23	38	60.5
**Comorbidities**			
HIV	1	28	3.6
Immunosuppressive treatment	6	29	20.7
Diabetes	4	30	13.3
Malignancy	1	28	3.6
CVD	0	28	0
Smoker	3	17	17.6
CKD	0	28	0
Pregnancy	5	36	13.9
**Clinical manifestation of TB**			
Isolated pulmonary	28	41	68.3
Extrapulmonary	1	41	2.4
Disseminated	12	41	29.3
**Specific organ manifestations**			
Lung	36	43	83.7
Pleura	4	38	10.5
Lymph node	3	38	7.9
Abdominal	8	38	21.1
Bone	4	38	10.5
Urogenital	2	38	5.3
CNS	5	38	13.2
Spine	3	38	7.9
Other	9	38	23.7
**Clinical manifestations**			
Fever	19	30	63.3
Weight loss	11	30	36.7
Night sweat	4	30	13.3
Cavity one lobe	7	33	21.2
Cavity bilobal	6	32	18.8
Infiltration one lobe	2	33	6.1
Infiltration bilobal	29	33	87.9
Haemoptysis	2	32	6.3
Coinfection	15	33	45.5
Bacterial pneumonia	10	15	66.7
UTI	2	15	13.3
Blood stream infection	2	15	13.3
Fungal	3	15	20.0
Viral	2	15	13.3
Other	1	15	6.7

*HIV* Human Immunodeficiency Virus, *CVD* cardiovascular disease, *CKD* chronic kidney disease, *TB* tuberculosis, *CNS* central nervous system, *UTI* urinary tract infection

Susceptibility testing of the mycobacteria was reported in 29 cases (67.4%). A total of 90.0% (27/29) of these patients had fully drug-sensitive tuberculosis (Table 2), while three patients had mono-resistant isolates (one isoniazid resistance, one rifampicin resistance and one streptomycin resistance). No cases of multidrug-resistant tuberculosis (MDR) or extensively drug-resistant tuberculosis (XDR) were reported. The use of tuberculostatic therapy was described in 34 of 38 cases (89.5%). Patients mainly received tuberculostatic standard therapy consisting of isoniazide, a rifamycin, ethambutol and pyrazinamide. Additional steroid therapy was administered in 15 of 23 cases (65.2%).

**Table 2:** Microbiological characteristics and medical treatment of patients

	**n**	**data available**	**[%]**
**Susceptibility pattern**			
DS	27	29	93.1
Monoresistance	3	29	10.3
INH Resistance	1	29	3.4
RMP Resistance	1	29	3.4
Streptomycin Resistance	1	29	3.4
MDR	0	29	0
XDR	0	29	0
**Antimycobacterial treatment**	34	38	89.5
INH	29	32	90.6
Rifamycin	30	32	93.8
Rifampicin	30	32	93.8
Rifabutin	1	32	3.1
Rifapentin	1	32	3.1
EMB	27	32	84.4
PZA	30	32	93.8
Fluoroquinolone	9	32	28.1
Levofloxacin	6	32	18.8
Ciprofloxacin	1	32	3.1
Moxifloxacin	2	32	6.3
Aminoglycoside			
Amikacin	4	32	12.5
Streptomycin	4	32	12.5
Linezolid	1	32	3.1
Cycloserin	1	32	3.1
**Adjunctive treatment**			
Steroids	15	23	65.2

*DS* Drug sensibility, *INH* Isoniazid, *RMP* Rifampicin, *MDR* multi drug resistant tuberculosis, *XDR* extensively drug-resistant tuberculosis, *EMB* Ethambutol, *PZA* Pyrazinamid

### Course of ECMO therapy

The majority of patients were described to suffer from ARDS (83.4%, 30/36) with a mean Horovitz quotient of 68.1 (IQR 30.0–131.0), while cardiac failure was described in 9/34 patients (26.1%) (Table 3). Twenty-seven out of 37 patients were exclusively on venovenous ECMO (VV V-V) (73%), while five patients were exclusively on venoarterial ECMO (VA V-A) (10.8%). In 4 additional patients, the type of ECMO was changed during the treatment process. All four of these patients were started on VA V-A due to respiratory failure coupled with hemodynamic instability.

**Table 3:** Parameters associated with ECMO treatment and outcome

	**n**	**data available**	**[%]**
Respiratory and circulatory parameters			
ARDS	30	36	83.4
Horovitz (mean, range)	68.1 (30.0–131.0)	16	
Cardiac failure	9	34	26.5
Catecholamine treatment	15	16	93.8
Course of ECMO treatment			
VV V-V^a^	31	37	83.7
VA V-A^a^	9	37	24.3
VVA VA-V^a^	2	37	5.4
VVA VV-A ^a^	1	37	2.7
LFPPV-ECCO_2_R	2	37	5.4
Implantation after diagnosis (days, median, IQR)	5 (1–12)	9	
Implantation after installation of TB treatment (days, median, IQR)	9 (2.5–63)	7	
Duration of ECMO therapy (days, median, IQR)	10.0 (7.0–28.0)	33	
Time of ventilation (days, median, IQR)	33 (11.5–57.0)	19	
Time in ICU (days, mean, range)	45.1 (12–114.0)	10	
Total observation time (days, median, IQR)	46.0 (10.8–250.9)	33	
Outcome			
Complications of ECMO therapy	17	35	48.6
Alveolar haemorrhage	4	17	23.5
Pneumothorax	2	17	11.7
Intracranial haemorrhage	2	17	11.7
Disconnection/Failure of pump	3	17	17.6
Thrombocytopenia	4	17	23.5
Bleeding other	5	17	29.4
Long term ventilation after ECMO necessary	2	28	7.1
Deceased	8	43	18.6
Deceased under ECMO therapy	7	43	16.3
Time to death (mean, IQR)	21.2 (12–39)	5	

*ARDS* acute respiratory distress syndrome, *IQR* interquartile range, *ECMO* extracorporeal membrane oxygenation, *VV V-V* venovenous ECMO, *VA V-A* venoarterial ECMO, *VVA VA-V* venoarterialvenous ECMO; *VVA VV-A* venovenoarterial ECMO, *LFPPV-ECCO*_*2*_*R* extracorporeal carbon dioxide removal, *TB* tuberculosis, *ICU* intensive care unit

^a^In four cases type of ECMO was changed during the treatment process

If mentioned, ECMO was implanted with a median delay of 9 days after initiation of TB treatment. ECMO treatment was continued for a median duration of 10 days, ranging from 3 to 89 days. The median time of ventilation was 33 days, ranging from 0 to 130 days (*n* = 19). Time in the ICU ranged from 12 to 114 days with a median of 45.1 days (*n* = 10). Patients were observed for up to 27 months.

Complications during ECMO therapy occurred in 17 of 35 cases (48.6%). The most common complications were bleeding complications and thrombocytopenia (29.4% (5/17) and 23.5% (4/17)). Alveolar haemorrhage (4/17; 23.5%) and intracranial haemorrhage (2/17; 11.7%) were also mentioned. Pneumothoraxes occurred in 11.7% (2/17) and disconnection of failure of the ECMO pump was mentioned in 17.6% (3/17). Two patients needed long term ventilation after ECMO weaning (7.1%). Eight patients died during the total observation time (18.6%), seven of whom died during ECMO therapy (16.3%) with a mean time to death of 21.1 days (Fig. 2B). Causes of death were intracranial haemorrhage, cardiac asystole or multiorgan failure. One patient died during ECMO installation due to a vascular complication. Individual patient characteristics are summarized in Table 4.

**Table 4:** Summary of included cases

Authors	PMID	Year	Gender	Age	Country of treatment	TB Manifestation	Drug Sensitivity	ARDS	Cardiac failure	ECMO type	ECMO days	Deceased
Abdul Samad et al.^a^	n/a	2022	f	n/a	India	Pulmonary	sensitive	n/a	n/a	n/a	n/a	no
Afolabi et al.^b^	n/a	2020	n/a	n/a	United Kingdom	Pulmonary	n/a	n/a	n/a	VV	n/a	no
Anand et al	**34975058**	2022	f	31	India	Pulmonary	sensitive	yes	no	VV	9	no
Andresen et al	**26029505**	2013	f	24	Chile	Pulmonary	sensitive	yes	no	ECCO2R—> VV	36	no
Araki et al	**35400697**	2022	m	26	Japan	Pulmonary	n/a	no	no	VV	5	no
Asif et al	n/a	2021	m	18	USA	Pulmonary	sensitive	yes	yes	VV	35	yes
Besa et al	**34341715**	2021	f	33	Chile	Pulmonary	sensitive	yes	no	VA—> VA-V—> VV	26	no
Bhardwaj et al	n/a	2016	f	24	USA	Pulmonary	n/a	yes	no	VV	8	no
Binh et al	**31341764**	2019	m	48	Vietnam	Pulmonary	sensitive	yes	no	VV	5	no
Castro et al	n/a	2014	f	0	Canada	Pulmonary	sensitive	no	no	VV	n/a	no
Charles et al	**22587731**	2013	f	65	France	Extrapulmonary	sensitive	no	yes	VA	10	no
Cogliandro et al.^c^	**24902570**	2014	m	20	Italy	Pulmonary	sensitive	yes	no	VV	89	no
Correa et al	**33537201**	2021	m	45	United Kingdom	Disseminated	sensitive	no	no	VV	7	no
Dosi et al	**32553326**	2020	f	40	India	Pulmonary	n/a	yes	yes	VV	8	yes
Frick et al	**26693121**	2015	f	33	Switzerland	Disseminated	sensitive	yes	no	VA—> VV	6	no
Haneke et al	**26498750**	2016	f	50	Germany	Pulmonary	sensitive	n/a	no	VV	21	no
Hauch et al	**33194891**	2020	m	0,6	Germany	Disseminated	sensitive	yes	no	VV	28	no
Homan et al	**1112137**	1975	f	58	USA	Disseminated	n/a	yes	yes	VA	6	yes
Hui et al	n/a	2021	n/a	n/a	China	Pulmonary	n/a	yes	n/a	n/a	n/a	yes
James et al	n/a	2014	m	31	USA	Pulmonary	sensitive	yes	no	VV	9	no
Kim et al.^d^	**24814840**	2014	f	44	South Korea	Pulmonary	sensitive	yes	no	VV	73	no
Lee et al	**28864904**	2017	m	24	South Korea	Pulmonary	sensitive	yes	yes	VA—> VV-A—> VV	24	no
Mauri et al.^c^	**21617600**	2012	m	20	Italy	Pulmonary	sensitive	yes	no	VV	89	no
Monier et al	**1421920**	1992	f	14	France	Disseminated	sensitive	yes	yes	LFPPV-ECCO2R	6	no
Nam et al.^d^	**26683127**	2016	f	44	South Korea	Pulmonary	sensitive	yes	no	VV	73	no
Omote al	**27408786**	2016	m	48	Japan	Pulmonary	sensitive	yes	no	VV	52	no
Park et al	**28583555**	2017	m	49	South Korea	Disseminated	n/a	yes	no	VA—> VA-V—> VV	11	no
Petrillo et al	**11761093**	2001	f	15	USA	Pulmonary	n/a	yes	no	VV	6	no
Quach et al	**37378441**	2021	m	0	USA	Pulmonary	sensitive	yes	yes	VV	13	no
Shang et al	**35910925**	2022	f	36	China	Disseminated	sensitive	yes	no	VV	7	no
Singh et al	n/a	2022	f	53	USA	Pulmonary	sensitive	no	no	n/a	0	yes
Snobre et al	**35086630**	2022	f	25	Belgium	Pulmonary	sensitive	yes	no	VV	10	no
Strunk et al	**26518065**	2016	m	42	Germany	Pulmonary	sensitive	yes	no	VV	n/a	yes
Tautz et al	**30417214**	2019	m	28	Germany	Disseminated	sensitive	yes	no	VV	53	no
Tiruvoipati et al	**17704305**	2007	f	10	United Kingdom	Disseminated	n/a	yes	no	VV	42	yes
Vesteinsdottir et al	**30662828**	2019	m	18	Iceland	Disseminated	sensitive	yes	no	VV	50	no
Wang et al.^e^	**34955089**	2022	f	n/a	China	n/a	n/a	yes	n/a	n/a	n/a	yes
Weisoly et al	**15095332**	2004	f	0	USA	Disseminated	sensitive	yes	yes	VA	3	no
Wu et al	**35899126**	2022	f	31	China	Pulmonary	sensitive	yes	no	VV	27	no
Yang et al	**34544354**	2021	m	40	China	Disseminated	RMP resistant	no	yes	VA	8	no

*f* female, *m* male, *USA* United States of America, *n/a* not available data, *ECMO* extracorporeal membrane oxygenation, *VV* venovenous ECMO, *VA* venoarterial ECMO; *VA-V* venoarterialvenous ECMO, *VV-A* venovenoarterial ECMO, *LFPPV-ECCO*_*2*_*R* extracorporeal carbon dioxide removal, references of all included articles are listed in the Supplementary material

^a^two patients included from the study

^b^four patients included from the study

^c^same patient

^d^same patient

^e^two patients included from the study

## Discussion

This meta-analysis demonstrates the feasibility of ECMO therapy in patients suffering from tuberculosis and respiratory or cardiac failure over a time period of 47 years. It appears that ECMO therapy in patients with TB could have a positive effect on overcoming ARDS or bridging the time until response to tuberculostatic therapy.

This study has two major limitations: first, although all available case reports in the medical literature have been included, the sample size remains comparatively low while reporting on a varied patient cohort, including newborns, children and adults with different courses of disease. Second, due to the relatively small sample size, the data presented here could be prone to publication bias and mortality could be underestimated. 

The long observation period (1975 – present) was chosen to comprise the entire period of ECMO therapy. In this time period, the technology and experience of ECMO therapy have completely changed, and implementation and survival rates have been increasing especially over the last decade. Therefore, the early reported cases might not be comparable to the present data. However, there are only two studies prior to 2000, which does not result in any significant impact in our analysis.

Despite these limitations, this study comprises prior experience with the use of ECMO therapy in this vulnerable population. A similar small cohort with limited data on ECMO application are HIV patients with severe ARDS caused by *Pneumocystis jirovecii* pneumonia (PJP). Rilinger et al. recommended that patients with HIV-associated PJP should not be withhold from ECMO therapy, while showing comparable results to those of our patient cohort [19]. Therefore, ECMO as a therapeutic tool in severe TB-associated ARDS should be considered and further implemented in the clinical routine. In particular, young TB patients without any other comorbidities have good potential for rehabilitation and therefore a very good chance of complete recovery. The average age of our cohort was lower than that of comparable ECMO cohorts. In 2018 Friedrichson et. al. found that half of the German cohort’s patients were over 45 years old [20]. Only 22.5% of patients in our cohort was older than 45 years.

In addition to improving gas exchange, ECMO is initiated to reduce ventilator-induced lung injury (VILI) and allows the facilitation of ultralow volume lung ventilation. ECMO therapy can be utilized to buy time and preserve organ function until TB medication can lessen the mycobacterial load. Due to the slow growth of mycobacteria, it can take weeks to observe any effects of tuberculostatic treatment. Additional coinfections such as bacterial pneumonia and fungal infections growing in the affected lung can further prolong the healing process. As in our cohort the rate of coinfection was relatively high, and their role as the etiology of ARDS in these patients cannot be excluded.

We did not identify any case reports of patients with MDR tuberculosis receiving ECMO therapy. The lack of published cases may be due to the lower share of MDR TB (4.2% in the EU in 2020) or even be partially caused by the fact that in many countries it is still common practice that patients with MDR-TB lung disease undergo early lobectomy or pneumectomy [21, 22]. In our view, multidrug-resistant tuberculosis should not be a contraindication for ECMO. MDR therapy might pose some additional issues since many medications are available only as oral formulations, and adequate absorption from the gastrointestinal tract might be impeded in the critically ill. Inadequate drug levels were also a problem for the first-line drug regimens as highlighted in three of the case reports [23–25]. In two cases rifampicin was switched from oral to intravenous administration to circumvent the issues with gastrointestinal absorption, but therapeutic levels were still not reached using the standard dose [24, 25]. In one case, rifampicin levels only increased after ECMO weaning leading the authors to indicate that the subtherapeutic levels might be caused by the larger volume of distribution during the ECMO circuit [24]. These findings suggest that TB patients need higher doses of standard medications during ECMO therapy even in the absence of dialysis, but more studies are needed to support this hypothesis.

## Conclusion

The practice of extracorporeal life support (ECLS) is becoming a crucial component of contemporary intensive therapy. Our systematic review and meta-analysis indicate that ECMO therapy is a feasible option for tuberculosis patients with respiratory or cardiac failure, bridging the time for pulmonary recovery. As the underlying disease is treatable, we advocate not to deprive this patient cohort of an evaluation for ECMO therapy. Additional prospective multicenter analyses are required to evaluate evidence-based guidance for clinical practice. 

### Supplementary Information

**Additional file 1. **

## Data Availability

The datasets used and analysed during the current study are available from the corresponding author upon reasonable request.

## Abbreviations

ARDS: Acute respiratory distress syndrome
ECC02R: Extracorporeal carbon dioxide removal
ECMO: Extracorporeal membrane oxygenation
LFPPV-ECCO2R: Low-frequency positive pressure ventilation with extracorporeal carbon dioxide removal
MDR TB: Multidrug resistant tuberculosis
NTM: Non-tuberculous mycobacteria
TB: Tuberculosis
VA V-A: Venoarterial ECMO
VV V-V: Venoveno ECMO
VVA V-VA: Venovenoarterial ECMO
VVA V-AV: Venoarterialveno ECMO
