# Supplementary material for: Extracorporeal membrane oxygenation (ECMO) in patients with tuberculosis: systematic review and meta-analysis of 43 cases
Source: BMC Pulm Med. 2024 Jan 22;24:47. doi: 10.1186/s12890-023-02715-x (PMC10801979; doi:10.1186/s12890-023-02715-x)
Supplement: Supplementary file 1 — Additional file 1. [file 12890_2023_2715_MOESM1_ESM.docx]

# Supplementary material 1

**References of included publications**

1. Abdul Samad M. Prevalence Miliary Tuberculosis During Pregnancy- A Cross Sectional Study in a Tertiary Hospital. Int J Pharm Clin Res 2022 146 391-398.

2. EuroELSO 2020 Poster Abstracts. Perfusion. 2020 May;35(1_suppl):93–282.

3. Anand S, Singla R, Kumar V, Dewan S, Faye A, Gupta A. A new role of extracorporeal membrane oxygenation in the management of tuberculosis with acute respiratory distress syndrome: A case report and review of literature. Lung India Off Organ Indian Chest Soc. 2022;39(1):77–9.

4. Andresen M, Tapia P, Mercado M, Bugedo G, Bravo S, Regueira T. Catastrophic respiratory failure from tuberculosis pneumonia: Survival after prolonged extracorporeal membrane oxygenation support. Respir Med Case Rep. 2013;10:19–22.

5. Araki T, Uehara N, Kamijo H, Suzuki Y, Komatsu M, Machida R, et al. Successful Rescue of Life-threatening Hemoptysis Caused by Pulmonary Tuberculosis Bridging with Extracorporeal Membrane Oxygenation. Intern Med Tokyo Jpn. 2022 Dec 1;61(23):3611–5.

6. Asif H, Bagatell S, Hodgin KF, Friedlander AL, Wood C. Reactivation MycobacteriumTuberculosis Associated Acute Respiratory Distress Syndrome Following COVID-19 Infection. In: TP47 TP047 COVID AND ARDS CASE REPORTS [Internet]. American Thoracic Society; 2021 [cited 2023 Oct 1]. p. A2454–A2454. Available from: https://www.atsjournals.org/doi/10.1164/ajrccm-conference.2021.203.1_MeetingAbstracts.A2454

7. Besa S, Morales ÁJ, Salas P, Bravo M S, Garrido-Olivares L. Extracorporeal membrane oxygenation for tuberculosis pneumonia with empyema. Respir Med Case Rep. 2021;34:101481.

8. Bhardwaj A, Matthias I, Bonk M, Malsin E, Lam J, Gutsche J, et al. 1938: PULMONARY TUBERCULOSIS-ASSOCIATED ARDS HAS A SIGNIFICANTLY HIGH MORTALITY: ECMO OFFERS HOPE. Crit Care Med. 2016 Dec;44(12):559–559.

9. Binh NG, Manabe T, Co DX, Thach PT, Tuan DQ, Cuong BV, et al. Tuberculosis-induced acute respiratory distress syndrome treated with veno-venous extracorporeal membrane oxygenation. Respir Med Case Rep. 2019;28:100900.

10. Castro. Congenital Pulmonary Tuberculosis In Twins. Am Thorac Soc 2014 Int Conf.

11. Charles P, Kahn JE, Ackermann F, Honderlick P, Lortholary O. Renal mucormycosis complicating extracorporeal membrane oxygenation. Med Mycol. 2013 Feb;51(2):193–5.

12. Cogliandro V, Lapadula G, Bandera A, Muscatello A, Marcolin R, Abbruzzese C, et al. ECMO: an alternative support for acute respiratory failure caused by tuberculosis? Int J Tuberc Lung Dis Off J Int Union Tuberc Lung Dis. 2014 Jul;18(7):879–81.

13. Correa G, Taylor D, Vogel D, Wyncoll D. A case of broncho-cutaneous fistula secondary to tuberculosis successfully managed with awake veno-venous extracorporeal membrane oxygenation. Respir Med Case Rep. 2021;32:101351.

14. Dosi R, Bhargava S, Khan P, Jain N, Jain G. An interesting case summary of tubercular pneumonia masquerading as viral pneumonia kept on extra-corporeal membrane oxygenation. Indian J Tuberc. 2020 Apr;67(2):268–73.

15. Frick SE, Flothmann C, Preiswerk B, Behr R, Genoni M. Extracorporeal Membrane Oxygenation in Miliary Tuberculosis and AIDS: A Case Report. Thorac Cardiovasc Surg Rep. 2015 Dec;4(1):18–20.

16. Haneke F, Schildhauer TA, Strauch J, Swol J. Use of extracorporeal membrane oxygenation in an awake patient after a major trauma with an incidental finding of tuberculosis. Perfusion. 2016 May;31(4):347–8.

17. Hauch H, Skrzypek S, Woessmann W, Lehmberg K, Ehl S, Speckmann C, et al. Tuberculosis-Associated HLH in an 8-Month-Old Infant: A Case Report and Review. Front Pediatr. 2020;8:556155.

18. Homan W, Harman E, Braun NM, Felton CP, King TK, Smith JP. Miliary tuberculosis presenting as acute respiratory failure: treatment by membrane oxygenator and ventricle pump. Chest. 1975 Mar;67(3):366–9.

19. Hui Y. Diagnosis and treatment of a patient with pneumoconiosis and tuberculosis complicated with severe COVID-19. 2021;(Journal of Environmental&Occupational Medicine ; 38(9):1029, 2021.).

20. James W, Brath L, Debesa O. Acute Respiratory Distress Syndrome Caused by Pulmonary Tuberculosis Successfully Managed With Extracorporeal Membrane Oxygenation. Chest. 2014 Oct;146(4):147A.

21. Kim HS, Lee ES, Cho YJ. Insufficient Serum Levels of Antituberculosis Agents During Venovenous Extracorporeal Membrane Oxygenation Therapy for Acute Respiratory Distress Syndrome in a Patient with Miliary Tuberculosis. ASAIO J. 2014 Jul;60(4):484–6.

22. Lee SI, Hwang HJ, Lee SY, Choi CH, Park CH, Park KY, et al. Veno-veno-arterial extracorporeal membrane oxygenation for acute respiratory distress syndrome with septic-induced cardiomyopathy due to severe pulmonary tuberculosis. J Artif Organs Off J Jpn Soc Artif Organs. 2017 Dec;20(4):359–64.

23. Mauri T, Foti G, Zanella A, Bombino M, Confalonieri A, Patroniti N, et al. Long-term extracorporeal membrane oxygenation with minimal ventilatory support: a new paradigm for severe ARDS? Minerva Anestesiol. 2012 Mar;78(3):385–9.

24. Monier B, Fauroux B, Chevalier JY, Leverger G, Nathanson M, Costil J, et al. Miliary tuberculosis with acute respiratory failure and histiocytic hemophagocytosis. Successful treatment with extracorporeal lung support and epipodophyllotoxin VP 16-213. Acta Paediatr Oslo Nor 1992. 1992 Sep;81(9):725–7.

25. Nam SJ, Cho YJ. The successful treatment of refractory respiratory failure due to miliary tuberculosis: survival after prolonged extracorporeal membrane oxygenation support. Clin Respir J. 2016 May;10(3):393–9.

26. Omote N, Kondoh Y, Taniguchi H, Kimura T, Kataoka K, Hasegawa R, et al. Acute respiratory distress syndrome due to severe pulmonary tuberculosis treated with extracorporeal membrane oxygenation: A case report and review of the literature. Respir Med Case Rep. 2016;19:31–3.

27. Park JI, Jung BH, Lee SG. Veno-Arterial-Venous Hybrid Mode of Extracorporeal Membrane Oxygenation for Acute Respiratory Distress Syndrome Combined With Septic Shock in a Liver Transplant Patient: A Case Report. Transplant Proc. 2017 Jun;49(5):1192–5.

28. Petrillo TM, Heard ML, Fortenberry JD, Stockwell JA, Leonard MK. Respiratory failure caused by tuberculous pneumonia requiring extracorporeal membrane oxygenation. Perfusion. 2001 Nov;16(6):525–9.

29. Quach HK, Scott BL, Lopez-Domowicz DA, Gambino RM, Evans AE, Ozment CP. Case Report: Congenital tuberculosis in a premature infant requiring extracorporeal membrane oxygenation. J Extra Corpor Technol. 2023 Jun;55(2):86–90.

30. Shang X, Zhang H, Chen S, Wang C, Lin M, Yu R. Diagnosis and ECMO Treatment of a Critically Ill Patient With Disseminated Mycobacterium tuberculosis: A Case Report. Front Public Health. 2022;10:938913.

31. Singh S, Houston S, Wallace A. Postintubation tracheal rupture associated with tuberculous tracheitis. Can J Respir Crit Care Sleep Med. 2023 Jul 4;7(4):213–4.

32. Snobre J, Payen MC, De Wit S. Severe pulmonary TB rescued with extracorporeal membrane oxygenation therapy. Int J Tuberc Lung Dis Off J Int Union Tuberc Lung Dis. 2022 Feb 1;26(2):169–70.

33. Strunk AK, Ciesek S, Schmidt JJ, Kühn C, Hoeper MM, Welte T, et al. Single- and multiple-dose pharmacokinetics of ethambutol and rifampicin in a tuberculosis patient with acute respiratory distress syndrome undergoing extended daily dialysis and ECMO treatment. Int J Infect Dis. 2016 Jan;42:1–3.

34. Tautz E, Wagner D, Wiesemann S, Jonaszik A, Bode C, Wengenmayer T, et al. Treatment of a broncho-esophageal fistula complicated by severe ARDS. Infection. 2019 Jun;47(3):483–7.

35. Tiruvoipati R, Balasubramanian SK, Entwisle JJ, Firmin RK, Peek GJ. Pseudocalcification on chest CT scan. Br J Radiol. 2007 Jul;80(955):e125-127.

36. Vesteinsdottir E, Myrdal G, Sverrisson KO, Skarphedinsdottir SJ, Gudlaugsson O, Karason S. ARDS from miliary tuberculosis successfully treated with ECMO. Respir Med Case Rep. 2019;26:165–7.

37. Wang K, Ren D, Qiu Z, Li W. Clinical analysis of pregnancy complicated with miliary tuberculosis. Ann Med. 2022 Dec;54(1):71–9.

38. Weisoly DL, Khan AM, Elidemir O, Smith KC. Congenital tuberculosis requiring extracorporeal membrane oxygenation. Pediatr Pulmonol. 2004 May;37(5):470–3.

39. Wu H, Tang Y, Xiong X, Zhu M, Yu H, Cheng D. Successful Application of Argatroban During VV-ECMO in a Pregnant Patient Complicated With ARDS due to Severe Tuberculosis: A Case Report and Literature Review. Front Pharmacol. 2022;13:866027.

40. Yang N, Zhou L, Mo X, Huang G, Wu P. Successful treatment of severe electrolyte imbalance-induced cardiac arrest caused by adrenal tuberculosis with ECMO in the ED. Int J Emerg Med. 2021 Sep 20;14(1):55.
